# Supplementary material for: The predicting formula and scoring system for cardiac iron overload for thalassaemia children: Study from a middle-income country
Source: PLoS One. 2024 Sep 4;19(9):e0309663. doi: 10.1371/journal.pone.0309663 (PMC11373841; doi:10.1371/journal.pone.0309663)
Supplement: S2 Checklist — (DOCX) [file pone.0309663.s002.docx]

STROBE Statement—checklist of items that should be included in reports of observational studies

|  | Item No. | Recommendation | Page  No. | Relevant text from manuscript |
| --- | --- | --- | --- | --- |
| **Title and abstract** | 1 | (*a*) Indicate the study’s design with a commonly used term in the title or the abstract | 25,68 | Cross sectional |
|  |  | (*b*) Provide in the abstract an informative and balanced summary of what was done and what was found | 25,32-46 | We recruited 80 children, of those, 8 (10%) were classified as cardiac iron overload based on MRI T2* screening.  The formula for cardiac iron overload was decided as 9.32 + 3.28 (Hb) + 2.9 (reticulocyte) + 3.64 (DT) + 4.1 (TR Vmax). A scoring system was defined by simplifying the formula. Values of Hb ≤ 8.2 g/L, reticulocyte ≤0.33%, DT ≤ 114.5 cm/s, and TR Vmax ≥ 2.37 m/s were given a score of 1, while others were assigned 0. Total scores of 0 or 1, 2, and 3 or 4 were categorized as low, moderate, and high risk for iron cardiac overload. |
| Introduction | | | |  |
| Background/rationale | 2 | Explain the scientific background and rationale for the investigation being reported | 22-24, 55-66 | In Indonesia, there has been an increase in the number of thalassaemia patients. In recent years, 9,009 (85.7%) out of 10,515 patients were aged 0–20 years in 2019 Early detection of cardiac iron overload using magnetic resonance imaging (MRI) T2* can prevent heart failure and death [5]. However, at present, MRI T2* is still limited in Indonesia due to its high cost as well as the lack of scanners required. Therefore, this study aims to develop a formula and scoring system for myocardial iron overload to improve management, prevent death, and increase life expectancy based on low-cost investigations. |
| Objectives | 3 | State specific objectives, including any prespecified hypotheses | 62-66 | To make a predicting formula and scoring system based on low-cost investigations  71-73 Thalassemia major aged 6-18 years with ferritin levels > 1,000 ng/mL were included.  This study recruited children aged > 6 years old who cooperated during the MRIT 2* procedure and ferritin level of > 1,000 ng/mL. As the majority of who those with ferritin level of > 1,000 ng/mL were at risk of cardiac involvement, left ventricular dilatation, abnormal contractility, tricuspid valve regurgitation, and pulmonary hypertension |
| Methods | | | |  |
| Study design | 4 | Present key elements of study design early in the paper | 25, 68 | Cross sectional |
| Setting | 5 | Describe the setting, locations, and relevant dates, including periods of recruitment, exposure, follow-up, and data collection | 68 | This cross sectional study was carried out at Rumah Sakit Anak dan Bunda Harapan Kita, Indonesia, during October 2017 to April 2019.  The clinical and demographic data included age, sex, age at first diagnosis and chelation, interval between transfusions, and type of chelation therapy were collected. |
| Participants | 6 | (*a*) *Cohort study*—Give the eligibility criteria, and the sources and methods of selection of participants. Describe methods of follow-up  *Case-control study*—Give the eligibility criteria, and the sources and methods of case ascertainment and control selection. Give the rationale for the choice of cases and controls  *Cross-sectional study*—Give the eligibility criteria, and the sources and methods of selection of participants | 68-89 | This cross sectional study was carried out Indonesia, from October 2017 to April 2019. Thalassemia major aged 6-18 years with ferritin levels > 1,000 ng/mL were included. The diagnosis of thalassemia major was based on Hb analysis. The clinical and demographic data included age, sex, age at first diagnosis and chelation, interval between transfusions, and type of chelation therapy were collected.  All subjects were scheduled for laboratory tests, ECG, left and right cardiac function, tricuspid valve regurgitation (TR Vmax), basal left ventricular and septal myocardial velocity with tissue doppler imaging (TDI) one week before blood transfusion. The laboratory test consisted of hemoglobin (Hb), reticulocyte, immature granulocyte (IG), WBC, platelets, urea, creatinine, AST, ALT, SI, and TIBC measurements |
|  |  | (*b*) *Cohort study*—For matched studies, give matching criteria and number of exposed and unexposed  *Case-control study*—For matched studies, give matching criteria and the number of controls per case |  |  |
| Variables |  | Clearly define all outcomes, exposures, predictors, potential confounders, and effect modifiers. Give diagnostic criteria, if applicable | 143-147 | The proportion of free variables in myocardium iron overload was assessed by performing a bivariate analysis between free and dependent variables. Receiver Operating Characteristics (ROC) procedure was carried out on all free variables. The results showed that Hb, reticulocyte, AST, ferritin, chelation adherence, DT, and TR Vmax had an AUC of > 0.60 |
| Data sources/ measurement | 8* | For each variable of interest, give sources of data and details of methods of assessment (measurement). Describe comparability of assessment methods if there is more than one group | 346 | Laboratory examination base on Pedoman Hasil Pemeriksaan Laboratorium Patologi Klinik Rumah Sakit Anak dan Bunda Harapan Kita 2014. Instalasi Laboratorium Terpadu,Jakarta;3-87, Indonesian.  Echocardiography and TDI were done based on Lopez L, Colan SD, Frommlet PC, Ensing GJ, Kendall K, Younoszai AK. Recommendations for quantification methods during the performance of a pediatric echocardiogam: a report from the Pediatric Measurements Writing Group of the American Society of Echocardiography Pediatric and Congenital Heart Disease Council. J Am Soc Echocardiog. 2010;23;465-95.  Ho CY, Solomon SD. A clinician’s guide to tissue Doppler imaging. Circulation. 2006;113:396−8. |
| Bias | 9 | Describe any efforts to address potential sources of bias | 85 | Echocardiograhy and TDI variables were done 3 time and calculated means |
| Study size | 10 | Explain how the study size was arrived at |  | Sample size was calculated with n _1_ = n_2_ (z_α_ √2PQ + z_β √_P_1_Q_1_ + P_2_Q_2_)^2^  (P_1_-P_2_)^2^  ^n1=n2=39^  There were also 8 free variables and multiplied by 10 (rule of thumb) |

Continued on next page

| Quantitative variables | 11 | Explain how quantitative variables were handled in the analyses. If applicable, describe which groupings were chosen and why | 74 | Chelation adherence was defined based on the ratio of total drug ingested and drug prescribe per month within the last three months.  There was was the lack of adherence to chelation therapy due to boredom. |
| --- | --- | --- | --- | --- |
| Statistical methods | 12 | (*a*) Describe all statistical methods, including those used to control for confounding | 94-97 | Multivariate logistic regression were presented as an adjusted Odds ratio (aOR) with a 95% confidence interval (CI). Significance was set as p<0,05.  The proportion of free variables in myocardium iron overload was assessed by performing a bivariate analysis between free and dependent variables. Receiver Operating Characteristics (ROC) procedure was carried out on all free variables. The results showed that Hb, reticulocyte, AST, ferritin, chelation adherence, DT, and TR Vmax had an AUC of > 0.60 Transformation of scoring system and risk classification of myocardial iron overload were also carried out |
|  |  | (*b*) Describe any methods used to examine subgroups and interactions |  |  |
|  |  | (*c*) Explain how missing data were addressed |  | We have No missing data found |
|  |  | (*d*) *Cohort study*—If applicable, explain how loss to follow-up was addressed  *Case-control study*—If applicable, explain how matching of cases and controls was addressed  *Cross-sectional study*—If applicable, describe analytical methods taking account of sampling strategy |  |  |
|  |  | (*e*) Describe any sensitivity analyses | 94-95 | Multivariate logistic regression were presented as an adjusted Odds ratio (aOR) with a 95% confidence interval (CI). Significance was set as p<0,05 |
| Results | | | | |
| Participants | 13* | (a) Report numbers of individuals at each stage of study—eg numbers potentially eligible, examined for eligibility, confirmed eligible, included in the study, completing follow-up, and analysed |  |  |
|  |  | (b) Give reasons for non-participation at each stage |  |  |
|  |  | (c) Consider use of a flow diagram |  | Thalassaemia age 6-18 years  Ferritin level > 1.000 ng/dl  One week before blood transfusion  Collecting clinical and demographic  age, sex, age at first diagnosis and chelation, interval between transfusions, type of chelation therapy were collected  All were scheduled for laboratory tests, ECG, left and right cardiac function, tricuspid valve regurgitation (TR Vmax), basal left ventricular and septal myocardial velocity with tissue Doppler imaging (TDI) |
| Descriptive data | 14* | (a) Give characteristics of study participants (eg demographic, clinical, social) and information on exposures and potential confounders |  | Thalassemia major aged 6-18 years with ferritin levels > 1,000 ng/mL were included. The diagnosis of thalassemia major was based on Hb analysis. The clinical and demographic data included age, sex, age at first diagnosis and chelation, interval between transfusions, and type of chelation therapy were collected. |
|  |  | (b) Indicate number of participants with missing data for each variable of interest |  | No missing data |
|  |  | (c) *Cohort study*—Summarise follow-up time (eg, average and total amount) |  |  |
| Outcome data | 15* | *Cohort study*—Report numbers of outcome events or summary measures over time |  |  |
|  |  | *Case-control study—*Report numbers in each exposure category, or summary measures of exposure |  |  |
|  |  | *Cross-sectional study—*Report numbers of outcome events or summary measures | 156  178  185 | 1. The Predicting formula for cardiac iron overload was -9.32 + 3.28 (Hb) + 2.79 (Reticulocyte) + 3 .64 (DT) + 4.10 (TR Vmax). 2. Transformation of scoring system for each variable category, the dichotomy value was Hb ≤8.2 g/dL=1 point and > 8.2 g/dL=0 point. Reticulocyte ≤0.33%=1 and >0.33= 0, DT ≤114.5 cm/s= 1 and >114.5 cm/s= 0, while TR Vmax ≥ 2.37 m/s = 1 point and < 2.37= 0 point. 3. Scoring system and risk classification for iron cardiac overload, scores of 0 or 1, 2, and 3 or 4 were categorized as low, moderate, and high-risk, respectively |
| Main results | 16 | (*a*) Give unadjusted estimates and, if applicable, confounder-adjusted estimates and their precision (eg, 95% confidence interval). Make clear which confounders were adjusted for and why they were included |  | multivariate logistic regression were presented as an adjusted Odds ratio (aOR) with a 95% confidence interval (CI). Significance was set as p<0,05. |
|  |  | (*b*) Report category boundaries when continuous variables were categorized |  |  |
|  |  | (*c*) If relevant, consider translating estimates of relative risk into absolute risk for a meaningful time period |  |  |

Continued on next page

| Other analyses | 17 | Report other analyses done—eg analyses of subgroups and interactions, and sensitivity analyses |  |  |
| --- | --- | --- | --- | --- |
| Discussion | | | | |
| Key results | 18 | Summarise key results with reference to study objectives | 48 | formula, cardiac iron overload; thalassemia |
| Limitations | 19 | Discuss limitations of the study, taking into account sources of potential bias or imprecision. Discuss both direction and magnitude of any potential bias | 225 | adherence method was calculated with history taking with no medical record support. This was one of limitation of this study. |
| Interpretation | 20 | Give a cautious overall interpretation of results considering objectives, limitations, multiplicity of analyses, results from similar studies, and other relevant evidence | 227-229 | However the adherence to chelation therapy variable was not play a rule on the predicting formula of myocardiac iron overload. |
| Generalisability | 21 | Discuss the generalisability (external validity) of the study results |  | The result of this investigation might be used to assess cardiac iron overload formula and scoring system in the absence of MRI T2* screening. |
| Other information | |  | | |
| Funding | 22 | Give the source of funding and the role of the funders for the present study and, if applicable, for the original study on which the present article is based |  | No external funding |

*Give information separately for cases and controls in case-control studies and, if applicable, for exposed and unexposed groups in cohort and cross-sectional studies.

**Note:** An Explanation and Elaboration article discusses each checklist item and gives methodological background and published examples of transparent reporting. The STROBE checklist is best used in conjunction with this article (freely available on the Web sites of PLoS Medicine at http://www.plosmedicine.org/, Annals of Internal Medicine at http://www.annals.org/, and Epidemiology at http://www.epidem.com/). Information on the STROBE Initiative is available at www.strobe-statement.org.
