# Supplementary material for: The predicting formula and scoring system for cardiac iron overload for thalassaemia children: Study from a middle-income country
Source: PLoS One. 2024 Sep 4;19(9):e0309663. doi: 10.1371/journal.pone.0309663 (PMC11373841; doi:10.1371/journal.pone.0309663)
Supplement: S2 File — (PDF) [file pone.0309663.s004.pdf]

Coordinates of the Curve

Test Result Variable(s): TRVmaxms

Positive if Gre	Sensitivity	Spesifisitas
-0,33	1	0
0,675	1	0,069
0,82	1	0,139
1,005	1	0,153
1,175	1	0,167
1,425	1	0,181
1,56	1	0,194
1,58	1	0,208
1,65	1	0,292
1,84	1	0,306
1,985	1	0,319
2,005	1	0,333
2,015	1	0,347
2,025	0,875	0,361
2,04	0,875	0,375
2,065	0,875	0,389
2,1	0,875	0,403
2,125	0,875	0,417
2,135	0,875	0,444
2,155	0,875	0,458
2,185	0,875	0,486
2,215	0,875	0,5
2,235	0,875	0,556
2,245	0,875	0,583
2,255	0,875	0,597

2,27	0,875	0,625
2,285	0,875	0,653
2,3	0,875	0,681
2,315	0,875	0,694
2,335	0,875	0,722
2,37	0,875	0,764
2,395	0,75	0,764
2,41	0,75	0,806
2,43	0,75	0,819
2,45	0,75	0,847
2,465	0,625	0,875
2,485	0,625	0,889
2,51	0,5	0,889
2,53	0,5	0,903
2,56	0,375	0,903
2,62	0,375	0,917
2,73	0,125	0,917
2,805	0	0,917
2,855	0	0,958
2,905	0	0,972
3,14	0	0,986
4,37	0	1

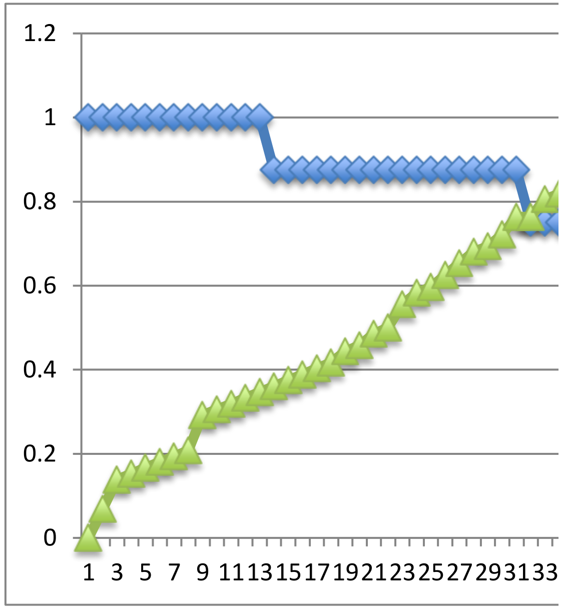

The test result variable(s): TRVmaxms has at least one tie between the positive actual state group and the negative actual state group.
a The smallest cutoff value is the minimum observed test value minus 1, and the largest cutoff value is the maximum observed test value

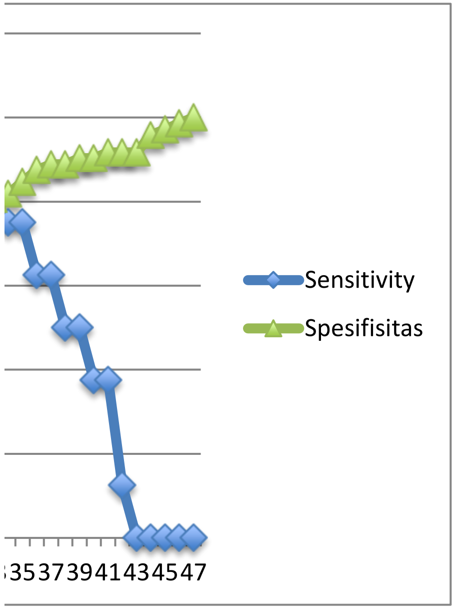

plus 1. All the other cutoff values are the averages of two consecutive ordered observed test values.
