# Supplementary material for: The predicting formula and scoring system for cardiac iron overload for thalassaemia children: Study from a middle-income country
Source: PLoS One. 2024 Sep 4;19(9):e0309663. doi: 10.1371/journal.pone.0309663 (PMC11373841; doi:10.1371/journal.pone.0309663)
Supplement: S3 File — (PDF) [file pone.0309663.s005.pdf]

Coordinates of the Curve

Test Result Variable(s): Hb

Positive if Gre	Sensitivity	Spesifisitas
4,5	1	0
6,1	0,986	0
6,95	0,972	0
7,3	0,972	0,125
7,45	0,944	0,25
7,55	0,944	0,375
7,7	0,917	0,375
7,9	0,903	0,5
8,05	0,861	0,5
8,25	0,861	0,625
8,45	0,819	0,625
8,55	0,792	0,625
8,65	0,722	0,625
8,75	0,694	0,625
8,85	0,653	0,625
8,95	0,597	0,625
9,1	0,542	0,75
9,25	0,5	0,75
9,35	0,458	0,75
9,45	0,403	0,75
9,55	0,333	0,75
9,65	0,319	0,75
9,75	0,278	0,75
9,85	0,264	0,75
9,95	0,167	0,75
10,1	0,153	0,75
10,25	0,125	0,75
10,45	0,111	0,75
10,7	0,111	0,875
10,9	0,083	0,875
11,05	0,042	0,875
11,35	0,028	0,875
11,65	0,014	0,875
11,95	0,014	1
13,2	0	1

The test result variable(s): Hb has at least one tie between the positive actual state group and th

a The smallest cutoff value is the minimum observed test value minus 1, and the largest cutoff v

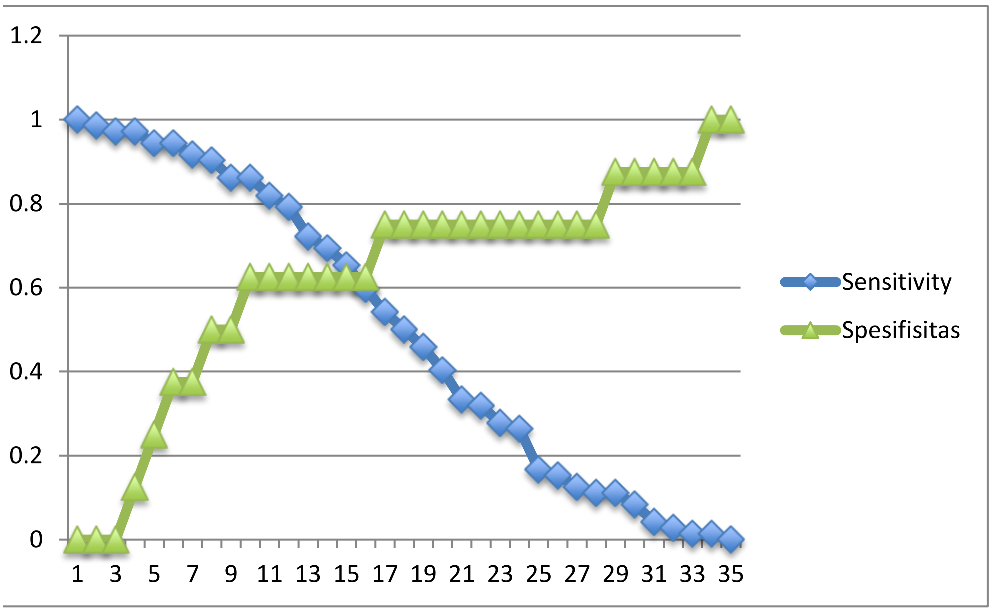

alue is the maximum observed test value plus 1. All the other cutoff values are the averages of tv

wo consecutive ordered observed test values.
