# Supplementary material for: The predicting formula and scoring system for cardiac iron overload for thalassaemia children: Study from a middle-income country
Source: PLoS One. 2024 Sep 4;19(9):e0309663. doi: 10.1371/journal.pone.0309663 (PMC11373841; doi:10.1371/journal.pone.0309663)
Supplement: Attachment — Submitted filename: Response to Reviewers.docx [file pone.0309663.s007.docx]

**Respons to Reviewers**

ACADEMIC EDITOR:

It is undoubtedly a very good paper, so I urge the authors to consider the indications of the reviewers, especially, to deepen the discussion and the projections of the article.
==============================

Please submit your revised manuscript by May 17 2024 11:59PM. If you will need more time than this to complete your revisions, please reply to this message or contact the journal office at [plosone@plos.org](mailto:plosone@plos.org). When you're ready to submit your revision, log on to https://www.editorialmanager.com/pone/ and select the 'Submissions Needing Revision' folder to locate your manuscript file.

Please include the following items when submitting your revised manuscript:

- A rebuttal letter that responds to each point raised by the academic editor and reviewer(s). You should upload this letter as a separate file labeled 'Response to Reviewers'.

Done

- A marked-up copy of your manuscript that highlights changes made to the original version. You should upload this as a separate file labeled 'Revised Manuscript with Track Changes'.

Done

- An unmarked version of your revised paper without tracked changes. You should upload this as a separate file labeled 'Manuscript'.

Done

If you would like to make changes to your financial disclosure, please include your updated statement in your cover letter. Guidelines for resubmitting your figure files are available below the reviewer comments at the end of this letter.

No changes on financial disclosure

If applicable, we recommend that you deposit your laboratory protocols in protocols.io to enhance the reproducibility of your results. Protocols.io assigns your protocol its own identifier (DOI) so that it can be cited independently in the future. For instructions see: <https://journals.plos.org/plosone/s/submission-guidelines#loc-laboratory-protocols>. Additionally, PLOS ONE offers an option for publishing peer-reviewed Lab Protocol articles, which describe protocols hosted on protocols.io. Read more information on sharing protocols at <https://plos.org/protocols?utm_medium=editorial-email&utm_source=authorletters&utm_campaign=protocols>.

Revision: laboratory protocols has been added

We look forward to receiving your revised manuscript.

Kind regards,

Juan Luis Castillo-Navarrete, Ph.D.
Academic Editor
PLOS ONE

Journal requirements:

1. When submitting your revision, we need you to address these additional requirements.

Please ensure that your manuscript meets PLOS ONE's style requirements, including those for file naming. The PLOS ONE style templates can be found at 
https://journals.plos.org/plosone/s/file?id=wjVg/PLOSOne_formatting_sample_main_body.pdf and 
<https://journals.plos.org/plosone/s/file?id=ba62/PLOSOne_formatting_sample_title_authors_affiliations.pdf>.

Revision: Abstract structure has been revised

2. Please include captions for your Supporting Information files at the end of your manuscript, and update any in-text citations to match accordingly. Please see our Supporting Information guidelines for more information: http://journals.plos.org/plosone/s/supporting-information.

3. Please review your reference list to ensure that it is complete and correct. If you have cited papers that have been retracted, please include the rationale for doing so in the manuscript text, or remove these references and replace them with relevant current references. Any changes to the reference list should be mentioned in the rebuttal letter that accompanies your revised manuscript. If you need to cite a retracted article, indicate the article’s retracted status in the References list and also include a citation and full reference for the retraction notice.

Reviewers' comments:
Reviewer's Responses to Questions

**Comments to the Author**

1. Is the manuscript technically sound, and do the data support the conclusions?

The manuscript must describe a technically sound piece of scientific research with data that supports the conclusions. Experiments must have been conducted rigorously, with appropriate controls, replication, and sample sizes. The conclusions must be drawn appropriately based on the data presented.

Reviewer #1: Yes

Reviewer #2: Yes

2. Has the statistical analysis been performed appropriately and rigorously?

Reviewer #1: Yes

Reviewer #2: Yes

3. Have the authors made all data underlying the findings in their manuscript fully available?

The [PLOS Data policy](http://www.plosone.org/static/policies.action#sharing) requires authors to make all data underlying the findings described in their manuscript fully available without restriction, with rare exception (please refer to the Data Availability Statement in the manuscript PDF file). The data should be provided as part of the manuscript or its supporting information, or deposited to a public repository. For example, in addition to summary statistics, the data points behind means, medians and variance measures should be available. If there are restrictions on publicly sharing data—e.g. participant privacy or use of data from a third party—those must be specified.

Reviewer #1: No

Reviewer #2: Yes

Revision: means data has been added

4. Is the manuscript presented in an intelligible fashion and written in standard English?

PLOS ONE does not copyedit accepted manuscripts, so the language in submitted articles must be clear, correct, and unambiguous. Any typographical or grammatical errors should be corrected at revision, so please note any specific errors here.

Reviewer #1: Yes

Reviewer #2: Yes

5. Review Comments to the Author

Please use the space provided to explain your answers to the questions above. You may also include additional comments for the author, including concerns about dual publication, research ethics, or publication ethics. (Please upload your review as an attachment if it exceeds 20,000 characters)

Reviewer #1: According to what was presented, conclusive data are obtained to estimate a score that correlates with iron overload. A correlation is also suggested between adherence to chelation treatment and number of transfusions, as it may bias the results.
Detailed demographic data is not found in the supplementary material. It is suggested to attach them to review the data tabulation.

Explanation: During study, the chelation adherence and number of transfusion data were taken with history taking as most subjects didn’t have adequate monitoring book so might be bias. However in this study the chelation adherence and number of transfusion were not a parameter for making a formula

Reviewer #2: Dear Authors,

I hope this letter finds you well. I am delighted to have the opportunity to review your exceptional research in the field of Thalassemia. Your findings are invaluable contributions to the scientific community and hold great significance for both researchers and patients alike. Please allow me to provide some constructive feedback aimed at enhancing the quality of your manuscript, organized according to each section:

1. Title: The title is informative and relevant, effectively capturing the essence of your study.

## Revision: The predicting formula and scoring system for cardiac iron overload for thalassaemia children: Study from a Middle-Income Country

Short title: A formula and scoring system for cardiac iron overload in thalassemia children

As a suggestion on Discussion and Conclusions below: Addressing the socioeconomic implications of your findings and the potential of the formula for early detection of cardiac iron overload in regions where MRI T2 is unavailable could broaden the impact of your work.

2. Abstract: I would kindly suggest mentioning the formula in the conclusion section of the abstract. Additionally, incorporating relevant keywords such as "MRI T2" could prove beneficial for indexing and discoverability purposes. Furthermore, I recommend replacing the term "thalassemia major" with the more encompassing "thalassemia" throughout the abstract. The remaining portions of the abstract effectively convey the study's findings and methodological approach.

Revision: I have put the formula in the conclusion section of the abstract. I have also replaced the term of thalassemia major with thalassemia throughout the abstract.

3. Introduction: While conciseness is appreciated, a more substantial introduction could better communicate the importance and necessity of your research. The research question could be more firmly grounded in the existing literature, necessitating the inclusion of more up-to-date studies and references, particularly recently published works such as those by Meloni et al. and Fedai et al.

Revision: to a better communicate the importance and necessity of the study, I have added some information regarding side effect of regular blood transfusion such as cardiac fibrosis and pulmonary hypertension on introduction. Regular blood transfusions are required to treat thalassemia and might cause iron overload, organ dysfunction, heart failure (HF), cardiac fibrosis, pulmonary hypertension and death [1,3,5].

I have revised literature:

no 31 with a recently published work which done by Sharma M, Burns AT, Yap K, Prior DL. The role of imaging in pulmonary hypertension. Cardiovasc Diagn Ther 2021; 11:859-80. No 32. Fraidenburg DR, Machado RF. Pulmonary hypertension associated with thalassemia syndromes. Ann N Y Acad Sci. 2016;1:127–39.

4. Methods: The subject selection process is clearly delineated. The variables are well-defined and measured appropriately, and the study methods are valid and reliable. The level of detail provided is sufficient to facilitate replication of the study by others.

No revision done

5. Results: The presentation of data could be enhanced through the judicious use of visual aids, such as charts and graphs, which would effectively communicate the key findings of your research. A Receiver Operating Characteristic (ROC) graph is highly recommended. Appropriate labeling of columns and rows in tables is appreciated, but I would suggest removing any horizontal lines to enhance readability.

Revision: a ROC graph have been added on the manuscript

6. Discussion and Conclusions: A more comprehensive discussion, exploring the results from multiple perspectives, would be beneficial. Addressing the socioeconomic implications of your findings and the potential of the formula for early detection of cardiac iron overload in regions where MRI T2 is unavailable could broaden the impact of your work. Generalizing your results to low- and middle-income countries, where the problem you have identified is prevalent, would further underscore the global relevance of your research. The conclusions are well-supported by the results and effectively address the aims of the study. Additionally, the limitations acknowledged present opportunities for future research endeavors.

Revision: Addressing the socioeconomic implication has been added to the manuscript as well as the possibility of using formula and score system for the others low and middle income countries

7. Overall: The study design is appropriate and effectively addresses the research aims, contributing novel insights to the existing body of knowledge on this topic.

I commend your exceptional work and look forward to the positive impact it will have on the scientific community and the lives of those affected by Thalassemia.

6. PLOS authors have the option to publish the peer review history of their article ([what does this mean?](https://journals.plos.org/plosone/s/editorial-and-peer-review-process#loc-peer-review-history)). If published, this will include your full peer review and any attached files.
